# MiR‐155 promotes interleukin‐1β‐induced chondrocyte apoptosis and catabolic activity by targeting PIK3R1‐mediated PI3K/Akt pathway

**DOI:** 10.1111/jcmm.15388

**Published:** 2020-06-20

**Authors:** Zhiyong Fan, Yinghui Liu, Zhengliang Shi, Kai Deng, Hua Zhang, Qiutong Li, Shuxing Cao, Shentai Li, Hongliang Zhang

**Affiliations:** ^1^ Department of Orthopaedic Surgery The Second Hospital of Hebei Medical University Shijiazhuang China; ^2^ Department of Infectious Disease The Third Hospital of Hebei Medical University Shijiazhuang China

**Keywords:** apoptosis, catabolic activity, chondrocyte, MiR‐155, osteoarthritis, PIK3R1

## Abstract

Osteoarthritis (OA) is a common joint disease characterized by progressive cartilage degradation, in which elevated chondrocyte apoptosis and catabolic activity play an important role. MicroRNA‐155 (miR‐155) has recently been shown to regulate apoptosis and catabolic activity in some pathological circumstances, yet, whether and how miR‐155 is associated with OA pathology remain unexplored. We report here that miR‐155 level is significantly up‐regulated in human OA cartilage biopsies and also in primary chondrocytes stimulated by interleukin‐1β (IL‐1β), a pivotal pro‐catabolic factor promoting cartilage degradation. Moreover, miR‐155 inhibition attenuates and its overexpression promotes IL‐1β‐induced apoptosis and catabolic activity in chondrocytes in vitro. We also demonstrate that the PIK3R1 (p85α regulatory subunit of phosphoinositide 3‐kinase (PI3K)) is a target of miR‐155 in chondrocytes, and more importantly, PIK3R1 restoration abrogates miR‐155 effects on chondrocyte apoptosis and catabolic activity. Mechanistically, PIK3R1 positively regulates the transduction of PI3K/Akt pathway, and a specific Akt inhibitor reverses miR‐155 effects on promoting chondrocyte apoptosis and catabolic activity, phenocopying the results obtained via PIK3R1 knockdown, hence establishing that miR‐155 promotes chondrocyte apoptosis and catabolic activity through targeting PIK3R1‐mediated PI3K/Akt pathway activation. Altogether, our study discovers novel roles and mechanisms of miR‐155 in regulating chondrocyte apoptosis and catabolic activity, providing an implication for therapeutically intervening cartilage degradation and OA progression.

## 1 INTRODUCTION

Osteoarthritis (OA) is the most common chronic whole‐joint disease featured by the progressive destruction of articular cartilage.^1^ Other structural alterations including those occurring in subchondral bone, ligaments and synovial membrane also frequently appear in OA.^2^ OA causes pain, deformity and reduction of motion^3^ and represents a leading cause of physical disability and also imposes a great socioeconomic burden globally, inflicting approximate 10% of the elderly population aged over 60 years.^4^, ^5^ The evidence‐based guidelines for OA treatment, such as prescribing capsaicin, cane and oral non‐steroidal anti‐inflammatory drugs, have been recommended by the OA Research Society International (OARSI),^6^ but, and no effective therapy is available to actually modify disease progression.^7^ This dilemma could be largely attributed to our limited understanding of the molecular mechanisms that underlie OA pathogenesis, which dismays the development of effective treatment approaches.^8^ OA pathogenesis is very complex and involves mechanical, inflammatory and metabolic factors that ultimately destruct the synovial joint.^2^ Although the augmented catabolic activity and the apoptosis of chondrocytes, the sole cell type residing this anatomic compartment to fulfil the cartilage development and cartilaginous extracellular matrix (ECM) maintenance, have been shown to play an important role in promoting articular cartilage degradation and OA pathogenesis,^9^, ^10^ much unidentified factors and signalling pathways regulating these pathogenic processes need to be unveiled, which holds potential significance to offer therapeutic targets for developing disease‐modifying therapies.

Mechanistically, in OA cartilage, the increased production of catabolic factors in chondrocytes, such as the matrix‐degrading enzymes, particularly the matrix metalloproteinase 3 (MMP3) and MMP13, is crucial for accelerating the destruction of core ECM molecules, including the collagen and aggrecan, leading to their functional loss that is closely related to OA progression.^11^, ^12^ Aside from this, the chondrocyte cell death through apoptosis also contributes to cartilage ECM degradation.^13^ Various extracellular catabolic factors, like the pro‐inflammatory cytokine interleukin (IL)‐1β, can provoke the degradation of ECM molecules and apoptosis in chondrocytes,^14^ and a growing body of evidence has shown that some microRNAs (miRNAs), a class of small and non‐coding RNAs that post‐transcriptionally suppress gene expression by targeting mRNAs,^15^ play a role in regulating these pathologic processes.^16^, ^17^ The epigenetic roles of miRNAs in OA pathogenesis have also been recognized recently,^18^, ^19^ shedding new light on the association between miRNAs with OA pathogenesis, and also evokes an interest to engage in further relevant studies.

MiR‐155 is a multifunctional miRNA playing key roles in numerous physiological and pathological processes, such as inflammatory disease and cancer.^20^, ^21^ In recent years, miR‐155 has been reported to regulate apoptosis in various cell types, including immunologic and cardiac cells.^22^, ^23^ Moreover, miR‐155 also affects catabolic activity in nucleus pulposus.^24^, ^25^ In the current study, we aimed to investigate whether miR‐155 regulates chondrocyte apoptosis and catabolic activity. We demonstrate that miR‐155 promotes IL‐1β‐induced chondrocyte apoptosis and catabolic activity through modulating the PI3K/Akt pathway by means of targeting and suppressing the expression of PIK3R1.

## 2 MATERIALS AND METHODS

### 2.1 OA cartilage collection

OA cartilage tissues tested in this study were collected from OA patients who underwent total knee joint replacement surgery in our hospital (n = 18). Normal cartilage tissues from 8 patients without OA who underwent the amputation were used as negative controls (n = 8), due to our limited accessibility to clinical resources. There is no statistical significance in clinical characteristics of age and gender between OA and control patients. The ethical approval was obtained from the Institutional Ethics Committee of The Second Hospital of Hebei Medical University, which conforms to the principles of the Declaration of Helsinki. The written informed consent was also obtained from each patient prior to study. After collection, OA cartilage tissues were examined using the Safranin O‐fast green staining to evaluate the cartilage deterioration, and the severity of histological changes was graded according to a modified Mankin scale.^26^ The control tissues were also examined histologically and confirmed without early pathologic OA changes.

### 2.2 Human chondrocyte isolation and culture

The isolation and following culture of human primary chondrocytes were performed as described previously.^27^ Briefly, normal human articular cartilage tissues were processed in accordance with the approved guidelines and then minced and digested using 0.2% collagenase II (Sigma) prepared in Dulbecco's modified Eagle's medium (DMEM, Invitrogen: Carlsbad, CA, USA). After filtration and centrifugation and washing with sterile phosphate‐buffered saline (PBS), the extracted chondrocytes were maintained in DMEM containing 10% foetal bovine serum (FBS, Invitrogen) at 37°C with 5% CO_2_ in a humidified incubator. Medium was replaced every two days. The first‐passage chondrocytes were obtained after two weeks. All experiments performed with primary chondrocytes were conducted within one week thereafter.

### 2.3 Cell treatment and transfection

Primary chondrocytes were cultured with the complete DMEM medium containing 0, 2, 5 or 10 µg/mL human recombinant IL‐1β (Invitrogen) for 24 hours to induce apoptosis and catabolic activity. To inhibit Akt pathway activation, primary chondrocytes were treated with 1 µM Akt inhibitor MK‐2206 (Selleck) for 24 hours. To manipulate the expression of miR‐155, primary chondrocytes were seeded in a 6‐well plate and then transfected with 30 nM miR‐155 mimic or 200 nM miR‐155 inhibitor (Ribobio, Guangzhou, China) through using Lipofectamine 3000 reagent (Invitrogen) according to the manufacturer's instructions. Meanwhile, equal amounts of miRNA negative mimic control and inhibitor control (Ribobio) were transfected as controls. The sequences of oligonucleotides referred to a previous report.^28^ After transfection for at least 24 hours, miR‐155 level was confirmed by qRT‐PCR analysis or chondrocytes were used for further studies. To silence or overexpress PIK3R1 expression in chondrocytes, siRNA targeting human *PIK3R1* (si*PIK3R1*) (GenePharma, Shanghai, China) or pcDNA‐*PIK3R1* vector was transfected into chondrocytes using Lipofectamine RNAiMAX (Invitrogen) or Lipofectamine 3000 reagent according to the manufacturer's protocols. siRNA targeting negative control (siNC) and pcDNA vector were used as controls. The efficacy of silencing or overexpression was confirmed at least 48 hours after transfection.

### 2.4 Quantitative real‐time PCR (qRT‐PCR) analysis

Total RNA from cartilage tissues and primary chondrocytes was extracted by using TRIzol reagent (Invitrogen: Carlsbad, CA, USA). Total RNA was reverse‐transcribed into cDNA with the PrimeScipt RT Master Mix Kit (Takara: Dalian, China) and SYBR PrimeScript miRNA RT‐PCR Kit (Takara: Dalian, China). cDNA levels were monitored by qRT‐PCR analysis on a 7500 Sequence Detection System (Applied Biosystems: Foster City, CA, USA) using gene‐specific primers (available when requested) and SYBR Premix Ex Taq (Takara: Dalian, China). Fold expression change was calculated by the comparative threshold cycle (Ct) with the formula 2^−ΔΔCt^ method. *RNU6B* and *GAPDH* were measured as endogenous controls for miRNA and mRNA, respectively.

### 2.5 Western blot analysis

Cartilage tissues and primary chondrocytes were lysed to obtain protein extracts, which were subjected to SDS‐PAGE (8%‐12% gel) and then transferred to polyvinylidene fluoride membranes (Millipore: Billerica, MA, USA). Membranes were blocked for 1 hour with 5% non‐fat milk prepared in Tris‐buffered saline (TBS) containing 0.1% Tween 20 (TBST) at room temperature (RT). Next, membranes were incubated overnight at 4°C with specific primary antibodies against PIK3R1 (Proteintech: Chicago, IL, USA, 60225‐1‐I, 1:500), cleaved caspase‐3 (Cell Signaling: Beverly, MA, USA, #9661, 1:1000), collagen Ⅱ (Novus Biologicals: Littleton, CO, USA, NBP1‐77795, 1:2000), aggrecan (abcam, ab3778, 1:1000), MMP3 (abcam: Cambridge, MA, USA, ab52915, 1:2000), MMP13 (abcam, ab39012, 1:2000) and GAPDH (Santa Cruz Biotechnology: Santa Cruz, CA, USA, sc‐32233, 1:5000). After washing with TBST, membranes were incubated with horseradish peroxidase‐labelled secondary antibodies at RT for 1 hour. The signal was visualized using an enhanced chemiluminescence reagent according to the manufacturer's protocol (Millipore: Billerica, MA, USA). GAPDH served as a loading control. The protein expression was quantified by ImageJ (http://rsb.info.nih.gov/ij/).

### 2.6 TdT mediated dUTP nick end labelling (TUNEL) assay

The apoptosis of chondrocytes was detected in situ through using a TUNEL assay according to the manufacturer's instructions (Roche). Briefly, primary chondrocytes plated on cover slides in a 6‐well plate were washed with PBS, fixed with 4% paraformaldehyde for 20 min and blocked with 5% bovine serum albumin (BSA) for 1 hour. Then, cover slides were immersed with TUNEL reaction mixture for 1 hour at 37°C and covered with fluorescence mounting medium (Zhongshan Golden Bridge Biotechnology, Beijing, China) in the darkness. Apoptotic cells were visualized under a microscope (LSM 510; Zeiss: Jena, Germany). Fifteen random fields in each group were analysed to calculate the percentage of TUENL‐positive cells (apoptotic).

### 2.7 Luciferase reporter assay

The wild‐type (wt) or mutant (mut) 3′‐UTR of human PIK3R1 containing potential binding sites for miR‐155 as predicted by the TargetScanHuman 7.2 (http://www.targetscan.org/vert_72/) was inserted into the pGL3‑luc vector (Promega: Madison, WI, USA) to obtain reporter plasmid. Primary chondrocytes were seeded in 96‐well plates and then cotransfected the reporter plasmid with miR‐155 mimic or miR‐155 inhibitor using Lipofectamine 3000 (Invitrogen). The negative control (NC) mimic and inhibitor were used as controls. At 36 hours after transfection, the luciferase activity was measured by a Dual‐Luciferase Reporter Assay System (Promega: Madison, WI, USA) according to the manufacturer's instructions. The ratio of firefly and Renilla luciferase activities in each well was calculated. Each treatment was performed in 5 replicates.

### 2.8 Statistical analysis

All data were presented as the means ± *SEM* from at least three independent assays. The statistical significance was calculated using the unpaired Student's *t* test or analysis of variance (ANOVA) test with GraphPad Prism 5 software. *P* < 0.05 was considered to be statistically significant.

## 3 RESULTS

### 3.1 MiR‐155 level is elevated in OA cartilage and IL‐1β‐treated chondrocytes

**FIGURE 1:**
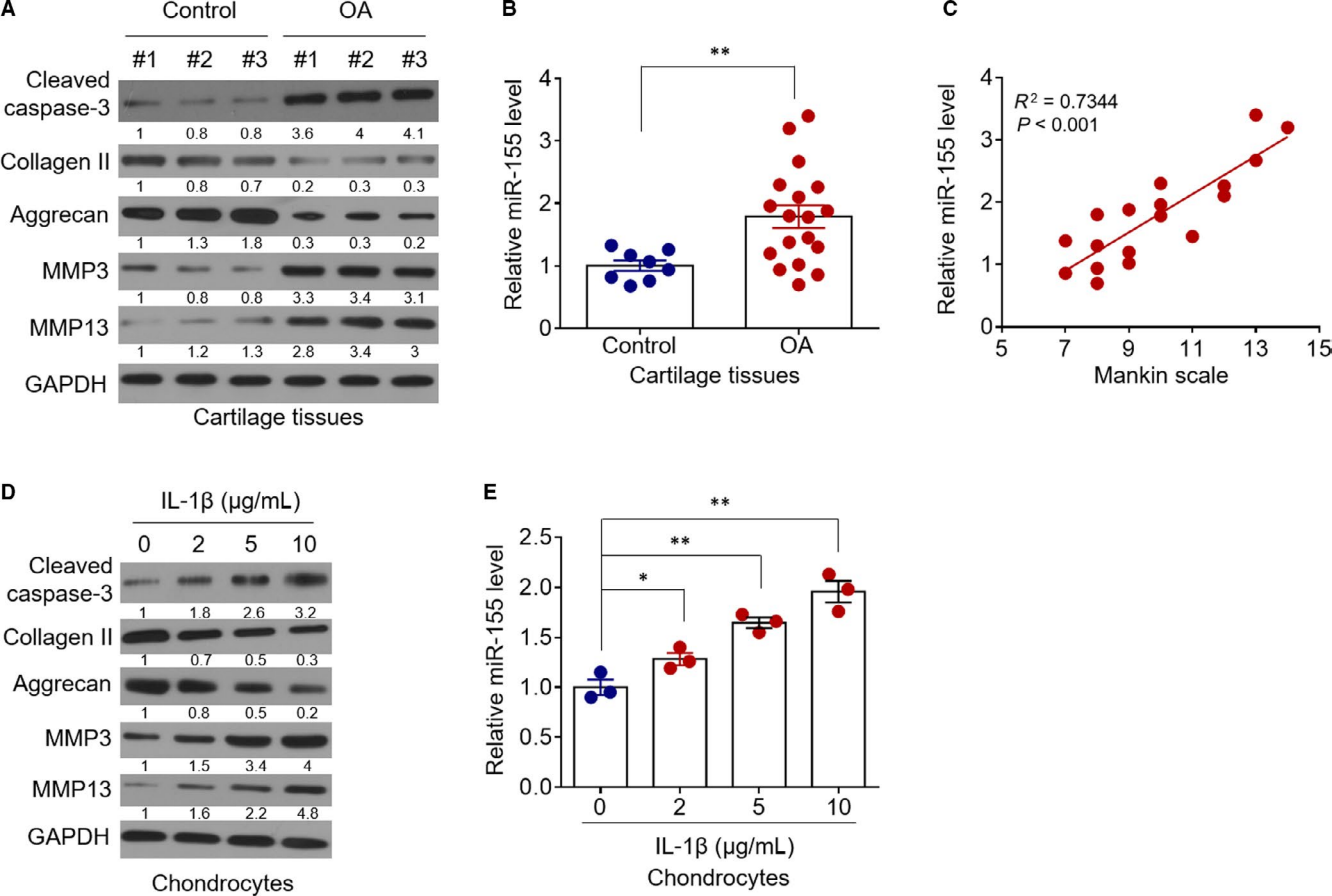
MiR‐155 is up‐regulated in OA cartilage and IL‐1β‐treated primary chondrocytes in vitro. A, Western blot analysis of cleaved caspase‐3, collagen Ⅱ, aggrecan, MMP3 and MMP13 expression in three representative cartilage tissues from healthy donors (control) and patients with osteoarthritis (OA). GAPDH is the loading control. Images represent three independent assays. Protein expression relative to GAPDH was quantified and noted beneath each band, and lane 1 was set with value 1. B, qRT‐PCR analysis of miR‐155 level in control group (n = 8) and OA group (n = 18). Data were normalized to *RNU6B* level*.* Each dot represents the mean value of each patient. Means ± *SEM* ANOVA test. C, Pearson's correlation analysis of miR‐155 level and a modified Mankin scale of 18 OA patients. *r* = 0.523, *P* < 0.001. D and E, Primary chondrocytes were treated with increasing concentrations of IL‐1β as indicated for 24 hours. The protein expression (D) and miR‐155 level (E) were analysed as in (A) and (B), respectively. Data are means ± *SEM* from 3 independent assays. ANOVA test, *P < 0.05; **P < 0.01

The OA articular cartilage tissues manifest signs of chondrocyte apoptosis, up‐regulation of matrix‐degrading enzymes and enhanced degradation of extracellular matrix (ECM).^10^, ^29^ Consistently, we also observed that compared with normal counterparts (n = 8), the expression levels of cleaved caspase‐3, a typical apoptosis marker, as well as the matrix metalloproteinase 3 (MMP3) and MMP13, were all markedly increased, and conversely, those of collagen Ⅱ and aggrecan were decreased in the articular cartilage tissues collected from the knee joint of OA patients (n = 18) (Figure 1A). To seek whether there exists a possible connection between miR‐155 and OA pathology, we initially checked whether miR‐155 expression is altered in OA articular cartilage tissues by applying the quantitative real‐time PCR (qRT‐PCR) analysis. As a result, we found that miR‐155 level was significantly up‐regulated in OA cartilage tissues in contrast with normal control samples (Figure 1B, *P* < 0.01). More pathologically relevant, miR‐155 level was positively correlated well with the severity of OA (Figure 1C), as graded by a modified Mankin scale,^26^ further implicating that miR‐155 may be involved in OA progression. The exposure of chondrocytes to a pro‐inflammatory cytokine interleukin (IL)‐1β can induce pathological phenotypes akin to those emerging during OA progression, such as increased apoptosis and catabolic activities.^30^, ^31^ These pathologic changes were reproducible in primary chondrocytes stimulated with IL‐1β in our study, as detected by the expression of corresponding molecular markers (Figure 1D). Noticeably, upon IL‐1β treatment, the expression level of miR‐155 in primary chondrocytes was drastically induced (Figure 1E) in a dose‐dependent manner, which is reminiscent of the result observed in OA cartilage tissues (Figure 1B). Together, these in vivo and in vitro data suggest that miR‐155 is associated with OA pathogenesis.

### 3.2 MiR‐155 functions to exaggerate IL‐1β‐induced chondrocyte apoptosis and catabolic activity in vitro

**FIGURE 2:**
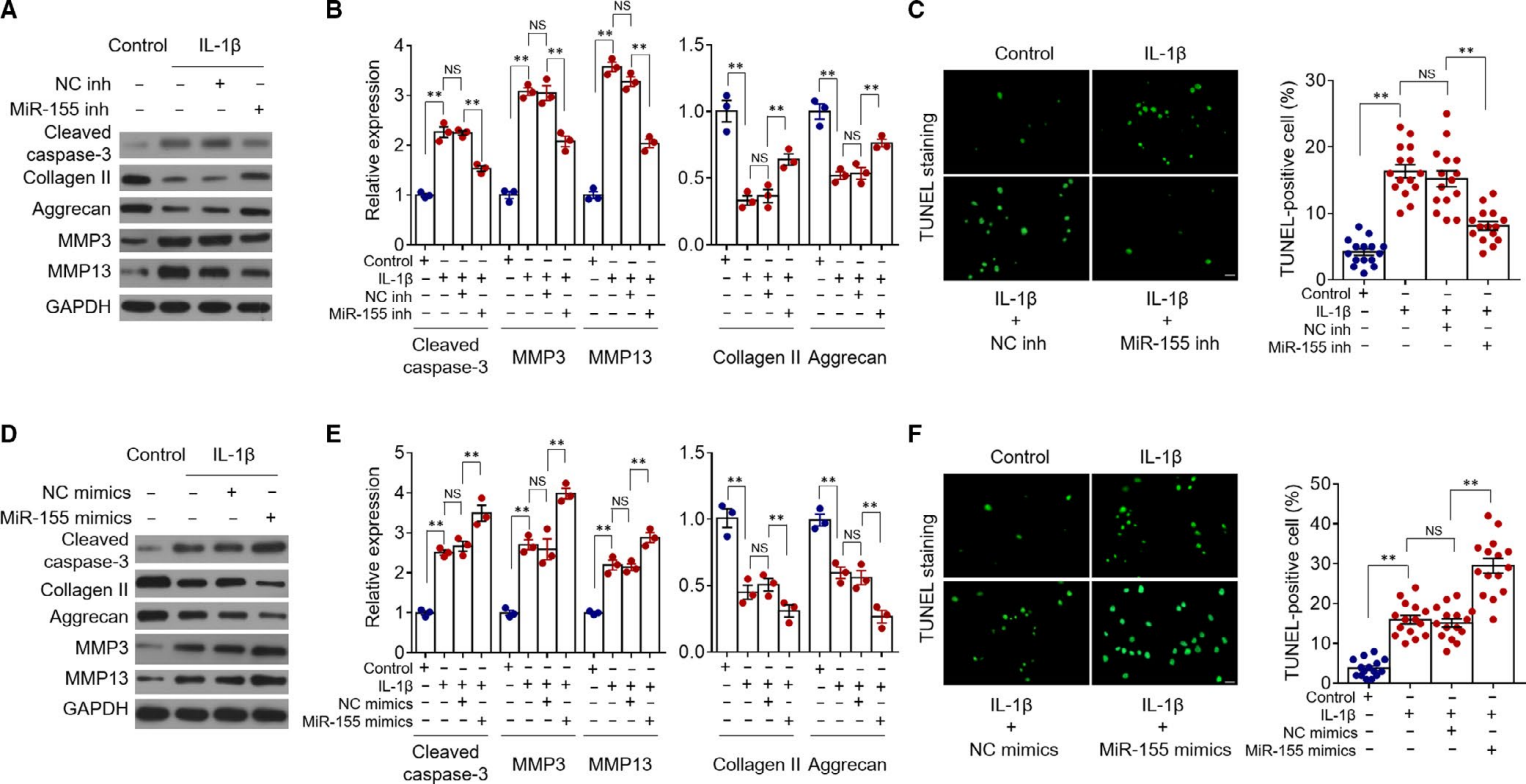
MiR‐155 promotes IL‐1β‐induced chondrocyte apoptosis and catabolic activity in vitro. A‐C, Primary chondrocytes were transfected with negative control (NC) inhibitors (NC inh) or miR‐155 inhibitors (miR‐155 inh) 24 hours prior to the treatment with or without 10 µg/mL IL‐1β for further 24 hours. A, The expression of proteins as indicated was determined by Western blot analysis. B, The relative protein expression to GAPDH was analysed by ImageJ software. Means ± *SEM* ANOVA test (n = 3), **P < 0.01. NS, not significant. C, Cell apoptosis was detected by TUNEL staining. The representative images (left, ᵡ 200 magnification) and quantification of TUNEL positive cells (right) are shown. Means ± *Sem* ANOVA test (n = 15), **P < 0.01. D‐F, Primary chondrocytes were transfected with NC mimics or miR‐155 mimics 24 hours prior to the treatment with or without 10 µg/mL IL‐1β for further 24 hours. The expression of proteins (D) and quantification (E) and cell apoptosis (F) were analysed as described in (A‐C). Means ± *SEM* ANOVA test, **P < 0.01. NS, not significant

Next, to interrogate the potential roles of miR‐155, we carried out loss‐of‐function studies and examined the effects on chondrocytes treated with IL‐1β. The inhibition of miR‐155 was accomplished via transfection of synthetic sequence‐specific oligonucleotides targeting miR‐155 (miR‐155 inhibitors).^28^ Consistent with Figure 1D, IL‐1β treatment caused obvious apoptosis and increased catabolic activity in primary chondrocytes, as demonstrated by elevated expression of cleaved caspase‐3 (Figure 2A,B), increased number of TUNEL‐positive cells (Figure 2C), up‐regulation of MMP3 and MMP13 and down‐regulation of collagen Ⅱ and aggrecan (Figure 2A,B). Functionally, under this treatment, compared with transfection of negative control oligonucleotides (NC inhibitors), miR‐155 inhibition notably reversed these pathological changes induced by IL‐1β (Figure 2A,C). In order to consolidate miR‐155 function, we then conducted gain‐of‐function studies to overexpress miR‐155 in chondrocytes through transfecting synthetic miRNA mimics (miR‐155 mimics). As shown, contrary to miR‐155 inhibition (Figure 2A,C), the overexpression of miR‐155 resulted in enhanced apoptosis (Figure 2D,F) and catabolic activity (Figure 2D,E) in chondrocytes under the treatment of IL‐1β. Thus, taken together, it could be concluded that miR‐155 functions as a positive regulator of IL‐1β‐induced chondrocyte apoptosis and catabolic activity, at least in vitro.

### 3.3 MiR‐155 targets PIK3R1 in chondrocytes

**FIGURE 3:**
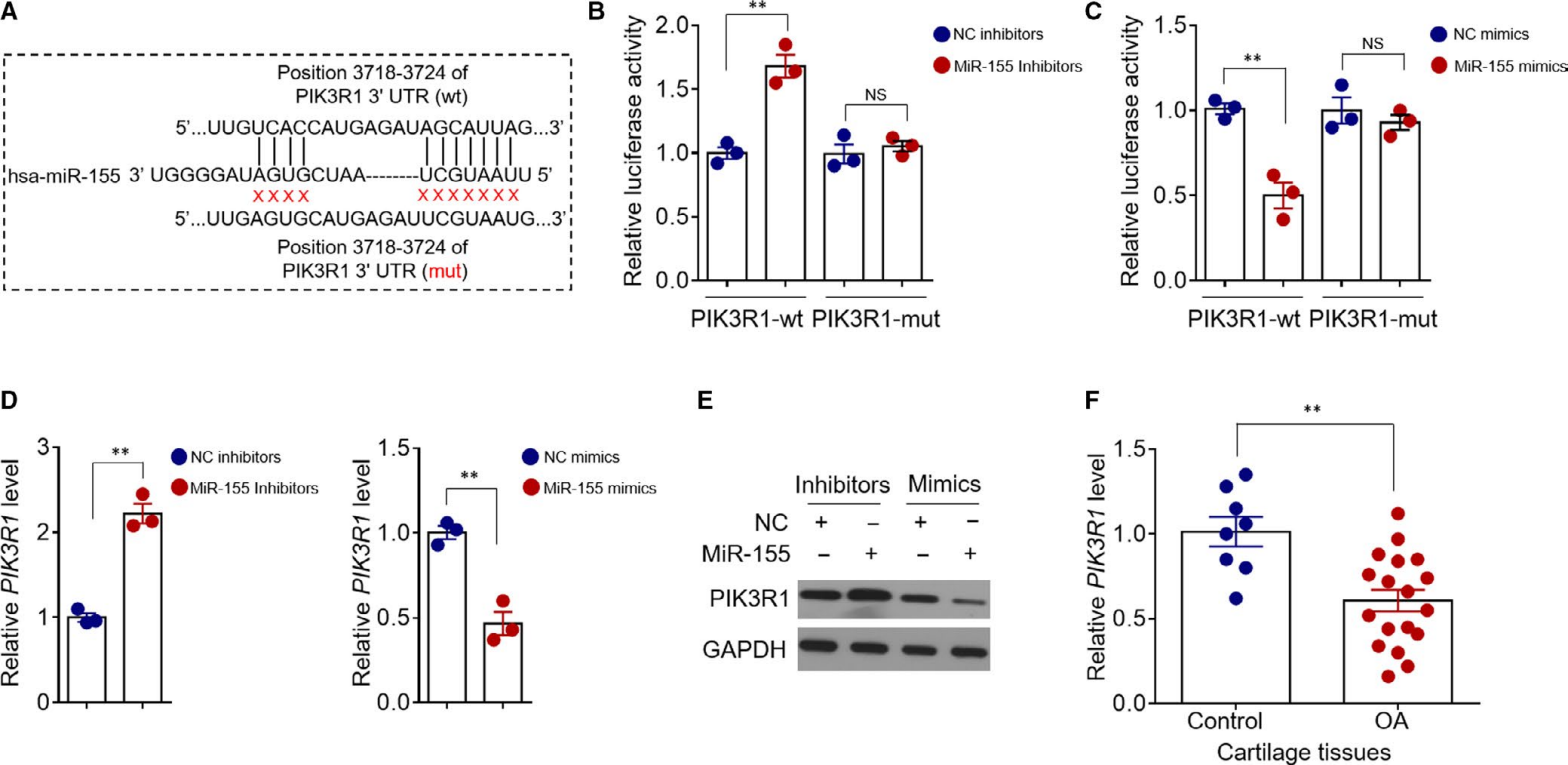
PIK3R1 is a direct target of miR‐155 in chondrocytes. A, The complementary sites among miR‐155 and the wild‐type (wt) 3'‐UTR of human *PIK3R1* were predicted by the TargetScanHuman 7.2. The mutant sequence (mut) is also shown. B, Primary chondrocytes were transfected with NC inhibitors or miR‐155 inhibitors along with pGL3‑luc vector plasmid containing wt or mut 3'‐UTR of human *PIK3R1*. The luciferase activity was determined at 36 hours post transfection. Means ± *SEM* Student's t test (n = 5), **P < 0.01. NS, not significant. C, Primary chondrocytes were transfected with NC mimics or miR‐155 mimics along with pGL3‑luc vector plasmid containing wt or mut 3'‐UTR of human *PIK3R1*. The luciferase activity was analysed as in (B). D and E, Primary chondrocytes were transfected individually with NC inhibitors, miR‐155 inhibitors, NC mimics and miR‐155 mimics as indicated for 48 hours. The mRNA level (D) and protein expression (E) of PIK3R1 were determined by qRT‐PCR analysis and Western blot analysis. Means ± *SEM* Student's *t* test (n = 5), **P < 0.01. F, qRT‐PCR analysis of PIK3R1 level in control group (n = 8) and OA group (n = 18). Data were normalized to *GAPDH* level*.* Each dot represents the mean value of each patient. Means ± *SEM* ANOVA test, **P < 0.01

MiRNAs perform versatile biological functions through suppressing their mRNA targets.^32^ To elucidate the underlying mechanism by which miR‐155 regulates chondrocyte apoptosis and catabolic activity, we sought its potential targets via an in silico algorithm (http://www.targetscan.org) (Figure 3A). Among these putative targets, the PIK3R1 (p85α regulatory subunit of phosphoinositide 3‐kinase (PI3K)) has been reported to impact chondrocyte survival and matrix synthesis.^33^ Hence, we asked whether PIK3R1 is a downstream target that mediates miR‐155 function. We first utilized luciferase reporter assay to confirm whether PIK3R1 is a *bona fide* target of miR‐155 in chondrocytes. As shown, MiR‐155 inhibition increased (Figure 3B), while its overexpression (Figure 3C) decreased the luciferase activity of luciferase construct carrying the wild‐type 3'‐UTR sequence of human *PIK3R1*. But, for the luciferase construct with mutant 3'‐UTR, no obvious effects were observed (Figure 3B,C). Moreover, miR‐155 inhibition in chondrocytes resulted in evident elevation of mRNA (Figure 3D) and protein (Figure 3E) levels of PIK3R1. In stark contrast, miR‐155 overexpression engendered remarkable PIK3R1 down‐regulation in chondrocytes (Figure 3D,E). These results clearly demonstrate that miR‐155 is able to target and suppress PIK3R1 expression in chondrocytes. As miR‐155 expression is up‐regulated in OA cartilage tissues (Figure 1B), we further conjectured that PIK3R1 may therefore be down‐regulated in these clinical samples. To address it, we detected the mRNA levels of PIK3R1 in control samples and OA cartilage tissues. Indeed, compared with control, a significant down‐regulation of PIK3R1 expression was observed in OA group (Figure 3F), providing an evidence showing the existence of miR‐155/PIK3R1 axis related to OA pathogenesis.

### 3.4 PIK3R1 restoration rescues miR‐155 effects on chondrocyte apoptosis and catabolic activity

**FIGURE 4:**
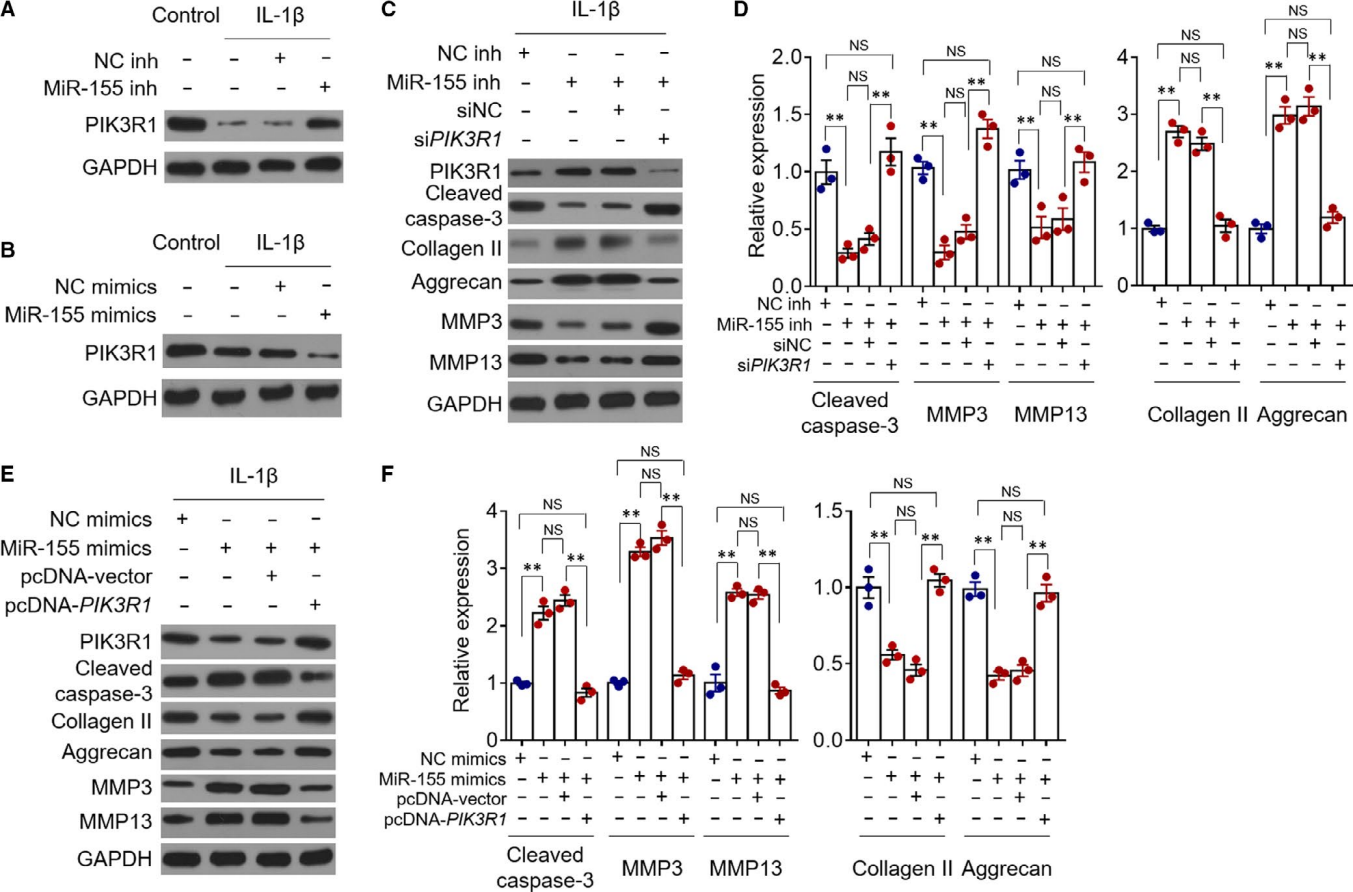
The targeted PIK3R1 mediates miR‐155 promotive effects on chondrocyte apoptosis and catabolic activity. A and B, Primary chondrocytes were transfected with NC inhibitors (NC inh) or miR‐155 inhibitors (miR‐155 inh) (A), or NC mimics or miR‐155 mimics (B), and cultured for further 24 hours in the presence or absence of 10 µg/mL IL‐1β. PIK3R1 expression was detected by Western blot analysis. Images represent three independent assays. C and D, Primary chondrocytes were transfected with NC inhibitors or miR‐155 inhibitors along with siRNA targeting negative control (siNC) or PIK3R1 (si*PIK3R1*) as indicated and cultured with 10 µg/mL IL‐1β for further 24 hours. The expression of PIK3R1, cleaved caspase‐3, collagen Ⅱ, aggrecan, MMP3 and MMP13 was analysed by Western blot assay. GAPDH is the loading control. C, Images represent three independent assays. D, The relative protein expression was analysed by ImageJ software. Means ± *SEM* ANOVA test (n = 3), **P < 0.01. NS, not significant. E and F, Primary chondrocytes were transfected with NC mimics or miR‐155 mimics along with pcDNA vector or pcDNA‐*PIK3R1* as indicated and cultured with 10 µg/mL IL‐1β for further 24 hours. The analysis of protein expression was performed as in (C‐D)

IL‐1β treatment decreased PIK3R1 level in chondrocytes (Figure 4A,B). Similar to those findings observed in unstimulated chondrocytes (Figure 3D,E), in IL‐1β‐treated chondrocytes, miR‐155 inhibition led to pronounced recovery of PIK3R1 expression (Figure 4A) and, in comparison, miR‐155 overexpression caused further decrease in PIK3R1 expression (Figure 4B), indicating that the negative regulation of PIK3R1 by miR‐155 is also effective under this condition. On this basis, we asked whether PIK3R1 is the mediator of miR‐155 function. To demonstrate this, we knocked down PIK3R1 expression in IL‐1β‐treated chondrocytes via the short interfering RNA (siRNA) technique. We found that upon siRNA‐mediated knockdown of PIK3R1, the effects of miR‐155 inhibition on chondrocyte apoptosis and catabolic activity, as indicated by marker expression detected by Western blot analysis, were completely abrogated (Figure 4C,D). Furthermore, the effects on chondrocyte apoptosis and catabolic activity imposed by miR‐155 overexpression were prominently reversed when PIK3R1 level was restored via transfecting pcDNA‐*PIK3R1* vector (Figure 4E,F). Collectively, these lines of evidence prove that miR‐155 promotes IL‐1β‐induced chondrocyte apoptosis and catabolic activity through targeting and suppressing the expression of PIK3R1.

### 3.5 MiR‐155 promotes chondrocyte apoptosis and catabolic activity by targeting PIK3R1‐mediated PI3K/Akt pathway

**FIGURE 5:**
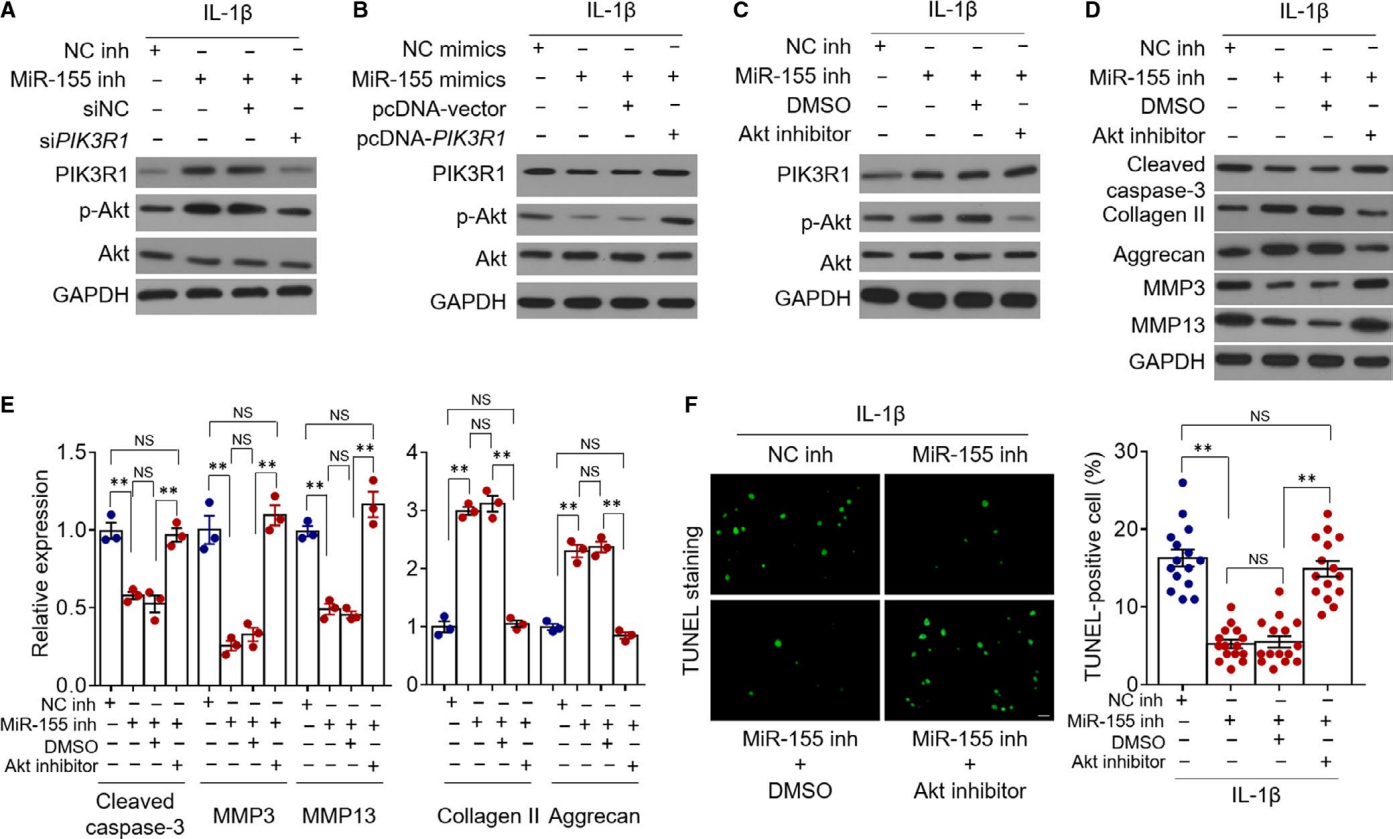
MiR‐155 promotes chondrocyte apoptosis and catabolic activity through targeting PIK3R1‐mediated PI3K/Akt pathway. A, Primary chondrocytes were transfected with NC inhibitors (NC inh) or miR‐155 inhibitors (miR‐155 inh) along with siNC or si*PIK3R1* as indicated and cultured with 10 µg/mL IL‐1β for further 24 hours. The expression of PIK3R1, p‐Akt and Akt was analysed by Western blot assay. GAPDH is the loading control. B, Primary chondrocytes were transfected with NC mimics or miR‐155 mimics along with pcDNA vector or pcDNA‐*PIK3R1* as indicated and cultured with 10 µg/mL IL‐1β for further 24 hours. The protein expression was analysed as in (A). C and E, Primary chondrocytes were transfected with NC inh or miR‐155 inh along with DMSO or 1 µM Akt inhibitor MK‐2206 as indicated and cultured with 10 µg/mL IL‐1β for further 24 hours. C, The protein expression of PIK3R1, p‐Akt and Akt was analysed as in (A). (D‐E) The protein expression of cleaved caspase‐3, collagen Ⅱ, aggrecan, MMP3 and MMP13 was also analysed. The representative images (D) were shown, and the relative protein expression (E) was analysed by ImageJ software. Means ± *SEM* ANOVA test (n = 3), **P < 0.01. NS, not significant. F, Primary chondrocytes were treated as in (C). Cell apoptosis was detected by TUNEL staining. The representative images (left, ᵡ 200 magnification) and quantification of TUNEL positive cells (right) are shown. Means ± *SEM* ANOVA test (n = 15), **P < 0.01. NS, not significant

**FIGURE 6:**
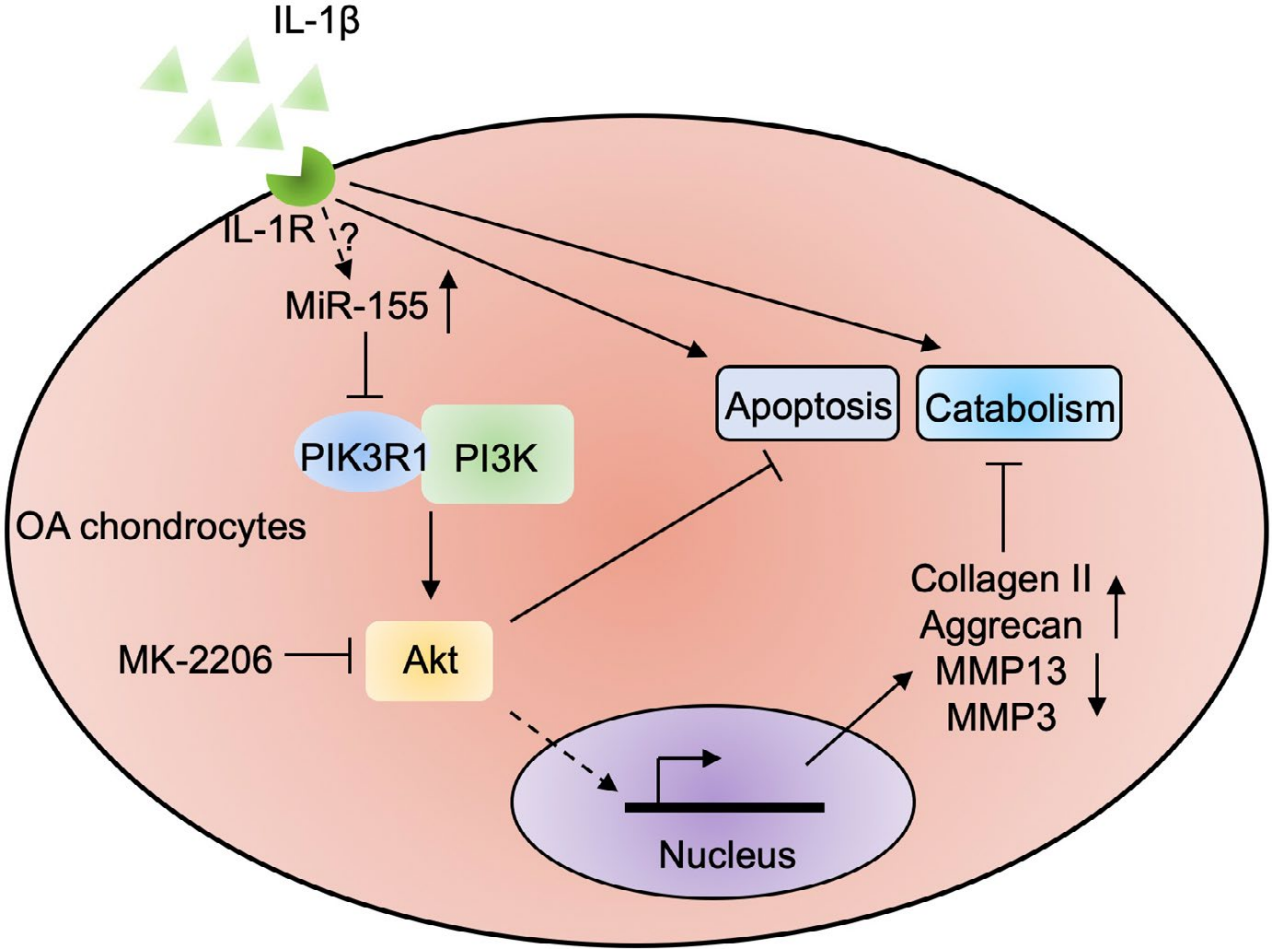
A schematic diagram of the mechanism underlies miR‐155 function in chondrocyte apoptosis and catabolic activity. In chondrocytes, the PI3K/Akt pathway normally functions to suppress apoptosis and catabolism once upon stimulation of IL‐1β through signalling transduction and indirect transcriptional control of collagen Ⅱ, aggrecan, MMP3 and MMP13. However, the IL‐1β secreted in the surroundings binds to its cognate receptor IL‐1R on the membrane of chondrocytes, leading to up‐regulation of miR‐155 via an unknown mechanism, which in turn inhibits PI3K/Akt pathway through targeting PIK3R1, one regulatory subunit of PI3K complex, and thus exacerbating IL‐1β adverse effects to induce chondrocyte apoptosis and catabolism

PIK3R1 is a regulatory subunit of the phosphoinositide 3‐kinase (PI3K), which generates phosphatidylinositol‐3‐phosphate (PIP3) upon activation via attaching to the plasma membrane receptors.^34^ Consequently, the downstream protein kinase B (Akt) binds to PIP3 and is then phosphorylated (p‐Akt) and activated.^34^ The PI3K/Akt pathway plays vital roles in several biological processes of chondrocytes, such as survival, differentiation, metabolism and inflammation.^35^, ^36^, ^37^ To elucidate the downstream PIK3R1‐associated signalling pathway that is responsible for determining miR‐155 function in chondrocyte apoptosis and catabolic activity, we concentrated on examining the possibility of PI3K/Akt pathway. First, we noticed that the activation status of PI3K/Akt pathway, as indicated by p‐Akt, was correspondingly altered along with the level of PIK3R1 in IL‐1β‐treated chondrocytes with miR‐155 inhibition (Figure 5A) or miR‐155 overexpression (Figure 5B), manifesting that PIK3R1 positively modulates PI3K/Akt pathway activation in this scenario. Subsequently, we treated chondrocytes with MK‐2206, a potent allosteric Akt inhibitor,^38^ to block the PI3K/Akt pathway activation which was induced by miR‐155 inhibition‐mediated PIK3R1 up‐regulation (Figure 5C). More importantly, Akt inhibition via MK‐2206 thoroughly diminished the effects of miR‐155 inhibition on restricting the apoptosis and catabolic activity in IL‐1β‐treated chondrocytes, as demonstrated by Western blot analysis (Figure 5D,E) and TUNEL assay (Figure 5F), photocopying the results obtained by PIK3R1 knockdown (Figure 4C,D). Overall, this study demonstrates that the PI3K/Akt pathway downstream of miR‐155/PIK3R1 axis dictates the cell fate of chondrocytes insulted with IL‐1β exposure (Figure 6), providing a mechanistic insight to understand miR‐155 roles in exacerbating IL‐1β‐induced chondrocyte apoptosis and catabolic activity.

## 4 DISSCUSSION

It is widely recognized that chondrocyte apoptosis and dysregulated catabolism, two key features in OA cartilage, contribute to disruption of cartilage homeostasis and ECM maintenance, leading to progressive tissue degeneration that underlies OA development and progression.^9^, ^39^ Therefore, preventing chondrocyte apoptosis and inhibiting catabolic activity appear to be promising therapeutic approaches for interfering OA deterioration.^40^, ^41^ In the current study, we reveal that miR‐155 acts as a positive regulator of apoptosis and catabolic activity in chondrocytes exposed to IL‐1β and also demonstrate that the targeted PIK3R1‐mediated PI3K/Akt pathway is the critical underlying molecular mechanism. Hence, according to these novel discoveries, we not only highlight the significant role of PI3K/Akt pathway in modulating chondrocyte apoptosis and catabolic activity, but also offer miR‐155 as a potential new molecular target for reducing chondrocyte apoptosis and cartilage catabolism in future OA therapy.

The features of increased apoptosis and catabolism in OA cartilage tissues were confirmed in clinical samples enrolled in our study. Meanwhile, miR‐155 level was also increased in these OA cartilage tissues and correlated positively with disease severity. These observations suggest that miR‐155 level is very possibly up‐regulated increasingly along with OA progression. Although this conclusion should be strengthened by further studies investigating more clinical samples, it may provide miR‐155 with a possible diagnostic value for disease evaluation. Coincidentally, as reported by a previous integrative omics profiling study, miR‐155 was shown as one of the highly‐up‐regulated miRNAs in OA cartilage tissues compared with normal counterparts.^42^ In addition, intriguingly, miR‐155 expression level was also found to be up‐regulated in OA chondrocytes compared with normal ones cultured in vitro.^43^ Consistently, two recent in vitro studies on human chondrocyte cultures have also revealed miR‐155 as a dysregulated miRNA among the expression profiles of miRNAs.^44^, ^45^ The mechanisms controlling miR‐155 expression are poorly known at present, but it can be up‐regulated by some inflammatory responses induced via lipopolysaccharide^46^ and cytokine IFN‐β.^47^ Increasing studies have shown that OA is an inflammatory disease, and chondrocytes and synovial cells overproduce multiple inflammatory mediators in focal sites, including IL‐1β.^1^ As we demonstrate that miR‐155 expression is also up‐regulated in IL‐1β‐treated chondrocytes in a dose‐dependent manner, we believe IL‐1β‐mediated inflammation at least partly contributes to miR‐155 up‐regulation in OA cartilage tissues. However, how exactly miR‐155 expression responds to IL‐1β stimulation at a molecular level in chondrocytes requires more in‐depth investigations.

The most well‐established roles of miR‐155 are its oncogenic activities documented in several types of cancers.^48^ Recently, miR‐155 has been shown to suppress autophagy in chondrocytes through inhibiting some key autophagy components, such as ULK1, ATG14 and ATG5.^49^ Autophagy is considered as a protective mechanism against OA development,^50^ and its activation reduces OA severity in experimental models.^51^ Presumably, miR‐155 may have adverse effects, if any, on OA pathology when autophagy is inhibited. In our study, we uncover a new function of miR‐155 in promoting apoptosis and catabolic activity in chondrocytes under a pathologic condition, ie IL‐1β exposure. Perhaps, we provide another clue indicating a detrimental role of miR‐155 in OA. Nevertheless, direct in vivo evidence is lacking and experiments performed with OA animal models are warranted in future. For instance, it would be intriguing to test whether miR‐155 inhibition in cartilage tissues is able to alleviate chondrocyte apoptosis and reduce cartilage degradation in animals with experimental OA. It also should be noted that we only conducted in vitro assays, and more elaborated techniques for detecting apoptosis and animal experiments would be necessary to strengthen our findings. Addressing this issue is helpful to advance our understanding of the physiological significance of miR‐155 in OA pathogenesis.

Previous studies have reported that PIK3R1 is a target of miR‐155 in diffuse large B cell lymphoma^52^ and breast cancer.^53^ In accordance with these, we also identified PIK3R1 as a direct target and suppressed by miR‐155 in chondrocytes. This regulation of PIK3R1 may exist in human cartilage tissues as well, as contrary to miR‐155 up‐regulation, PIK3R1 expression exhibits decrease in OA cartilage, which implies that aside from miR‐155, PIK3R1 may also be involved in OA pathogenesis. This is very possible, because *PIK3R1* gene loci were identified to be associated with hip OA.^54^, ^55^ PIK3R1 functions as a positive regulatory subunit for PI3K.^56^ The PI3K/Akt pathway promotes chondrocyte survival and ECM synthesis, and its activation ameliorates cartilage damage.^33^ We found that both PIK3R1 knockdown and pharmaceutical inhibition of PI3K/Akt pathway activation could rescue miR‐155 effects on IL‐1β‐induced chondrocyte apoptosis and catabolic activity, thus establishing that the targeted PIK3R1‐mediated PI3K/Akt pathway by miR‐155 is the downstream signalling that modulates chondrocyte survival and catabolism in response to IL‐1β insults.

## 5 CONCLUSION

Taken together, our study establishes an unanticipated role and mechanism of miR‐155 in chondrocyte apoptosis and catabolism with in vitro investigations, which may provide a molecular basis for targeting the miR‐155/PIK3R1 axis and PI3K/Akt pathway in intervening OA progression. Relevant in vivo studies performed with OA animal models are required to collaborate our findings, which would provide more direct and physiological connection between miR‐155 and OA pathology.

## CONFLICT OF INTEREST

None declared.

## AUTHOR CONTRIBUTION

Zhiyong Fan and Yinghui Liu designed and performed experiments, and analysed data. Zhengliang Shi, Kai Deng, Qiutong Li, Shuxing Cao, Shentai Li and Hongliang Zhang performed experiments and analysed data. Zhiyong Fan and Hua Zhang designed experiments, provided materials and wrote the manuscript.

## Data Availability

The data that support the findings of this study are available from the corresponding author upon reasonable request.
